# Commentary: A Hypothesis for Examining Skeletal Muscle Biopsy-Derived Sarcolemmal nNOSμ as Surrogate for Enteric nNOSα Function

**DOI:** 10.3389/fmed.2015.00070

**Published:** 2015-09-30

**Authors:** Justin Percival

**Affiliations:** ^1^Department of Molecular and Cellular Pharmacology, University of Miami Miller School of Medicine, Miami, FL, USA

**Keywords:** nNOS, nitric oxide, skeletal muscle, nNOS splice variants, sarcolemmal nNOS, NOS1

Dr. Chaudhury puts forward an interesting hypothesis that reductions in sarcolemmal nNOSμ might parallel reductions in enteric neuron nNOSα expression, localization, and activity (1). In other words, nNOSμ expressed at the plasma membrane (sarcolemma) of skeletal muscle cells could act as a surrogate for nNOSα function. This possibility is attractive because sarcolemmal nNOSμ localization and expression can be readily assessed from peripheral skeletal muscle biopsies. Also, this possibility is enticing because it circumvents the difficulties of obtaining gastrointestinal tract biopsies to evaluate nNOSα dysregulation as a potential causal factor of gut dysfunction. We aim to advance the dialog on this issue by addressing key assumptions of this hypothesis from the point of view of skeletal muscle nNOS.

Key assumptions of this hypothesis include (1) the molecular similarity of the nNOSα and nNOSμ splice variants; (2) the mechanisms regulating nNOSα localization in neurons are similar to those regulating nNOSμ in skeletal muscle cells. The molecular similarity assumption is reasonably based on high protein homology between nNOSα and nNOSμ. Muscle nNOSμ contains an internal 34 amino acid insert in the autoregulatory domain not found in nNOSα (2). And while the study of isozyme-specific differences between nNOS splice variants is in its infancy, early studies suggest that μ insert inclusion reduces the speed of electron transfer from reductase to oxidase domains (3). The physiological significance of this altered catalytic activity of nNOSμ relative to nNOSα remains to be determined. From an evolutionary point of view, there has been positive selective pressure to retain both nNOSα and nNOSμ splice variants strongly suggesting key functional differences that confer advantage. Therefore, caution must be exercised to avoid overestimating the similarities of nNOSα and nNOSμ.

In addition, it is important not to overlook additional differences at the transcript level between nNOSα and nNOSμ, particularly in humans, due to alternative amino terminal exon choice that do not impact protein sequence. This provides additional molecular differences between nNOSα and nNOSμ and suggests potential mechanisms of differential control between nNOS splice variants-particularly between neurons and skeletal muscle cells (4). Transcript diversity represents an important regulatory mechanism for the control of tissue-specific distribution and function of *NOS1* gene splice variants and is an important consideration for understanding nNOS isozyme function salient to Dr. Chaudhury’s hypothesis.

The second key assumption is that similar mechanisms act to localize nNOSα in neurons and nNOSμ in skeletal muscle cells. Before discussing this point, it is worth describing the multiple spatially and functionally distinct pools of nNOS in skeletal muscle cells. Two functionally and spatially distinct nNOS splice variants are co-expressed in skeletal muscle cells-nNOSβ and nNOSμ (5, 6). We consider nNOSμ only here. Although the sarcolemmal localization of nNOSμ is well recognized, it is commonly overlooked that sarcolemmal nNOSμ represents not more than half of total nNOSμ expressed in skeletal muscle cells. Furthermore, a small fraction of that sarcolemmal nNOSμ resides on the postsynaptic membranes of skeletal muscle cells at the neuromuscular synapse. In addition, approximately half of muscle nNOSμ is localized to the cytosol where it regulates RYR1-mediated calcium release from the sarcoplasmic reticulum (7). Importantly, cytosolic nNOSμ is quite active in resting muscles; arguing against the proposition that nNOSμ requires localization to the sarcolemma to be active. This is not to say that the localization nNOSμ is not critical for some its functions in skeletal muscle, such as the attenuation of sympathetic vasoconstriction during exercise (8). We are saying that in skeletal muscle, nNOSμ does not have to be at the plasma membrane to be active and that nNOSμ has important non-sarcolemmal functions. Perhaps most importantly, the pool of nNOSμ at the neuromuscular junction in skeletal muscle may be the most relevant for comparison with enteric neuron nNOSα.

The localization of sarcolemmal nNOSμ is relatively well understood. In skeletal muscle, α-syntrophin is a critical and necessary scaffold for nNOSμ at the sarcolemma and neuromuscular synapse. By contrast, post synaptic density 95 (PSD-95) protein is the key scaffold in different neuronal cells of the central nervous system (9). Therefore, there is a critical difference in mechanisms used to localize plasma membrane-associated nNOS in neurons and skeletal muscle cells. This of course undermines the strength of the second key assumption of the hypothesis that the mechanisms localizing nNOS at the plasma membrane in skeletal muscle cells in neurons are similar enough.

However, of greater relevance and importance to the hypothesis is that the sarcolemmal localization of nNOSμ and its expression in skeletal muscle is dynamic [reviewed in Ref. (6, 10)]. The expression of nNOSμ and its association with the sarcolemma positively correlates with activity or exercise level in rodents and humans and may be a useful biomarker of exercise capacity. With greater activity, typically endurance type exercise, there is an increased expression of nNOSμ at the sarcolemma. Conversely, with a decrease in activity associated with conditions such as myopathy, chronic bedrest, or hindlimb unloading, nNOSμ translocates from the plasma membrane to the cytosol and can initiate a muscle wasting atrogene program by mechanisms that remain to be fully deciphered (10). In other words, nNOSμ appears to participate in a “use it or lose it” type mechanism to control muscle mass and exercise capacity. The molecular details underpinning this mechanism remain to be fully deciphered. However, this muscle-specific activity-based control of nNOSμ may confound the use of sarcolemmal nNOSμ as a proxy for enteric neuron nNOSα. It is quite likely that patients with severe gastrointestinal motility disorders will be more sedentary and perhaps exhibit reduced exercise tolerance, particularly if they lack normal nNOSμ activity. Therefore, as a secondary downstream consequence we would expect their skeletal muscles to express less sarcolemmal nNOSμ. In this scenario, these patients would always have less sarcolemmal nNOSμ. In other words, nNOSμ levels would reflect patient activity levels and not neuronal nNOSα making the evaluation of sarcolemmal nNOSμ uninformative as a surrogate for nNOSα in the gastrointestinal tract.

## Conflict of Interest Statement

The author declares that the research was conducted in the absence of any commercial or financial relationships that could be construed as a potential conflict of interest.

## Funding

JMP is supported by NIAMS (1R03AR066805-01) and the Department of Defense (MD140021). The content is solely the responsibility of the author and does not necessarily reflect the official views of NIAMS or Department of Defense.
